# Progress in Ophthalmology

**Published:** 1903-05-09

**Authors:** 


*(To be continued.)*

### Progress in Ophthalmology.

**The Etiology and Prevention of Senile Cataract.**—Jervey, of Greenville, S.C.,[1] has from the observation of one case brought forward a new theory as to the causation of cataract which may or may not be right, as it is always doubtful to go on the evidence of one case, but still it is an ingenious one and very plausible even if not true. The case is as follows. A man of 40 years of age was struck in his right eye with a piece of lime; this took place 20 years ago. The only remains of the accident were a paralysis of the sphincter pupillæ, which has existed since the injury. The pupil is 5 m.m. or 6 m.m. in diameter, regular, reacting easily to atropine but unresponsive to eserine. There is no reaction to light or accommotion. The anisocoria is very marked as the pupil of the left eye is normal. Vision in the right eye is rather faulty 5-100, and in the left 5-70. The vision in the right eye has been amblyopic since the accident. The vision of the left eye is failing. Equatorial radial opacities are faintly manifest in the lens cortex of this uninjured eye, but in the eye with the paralysed sphincter all the media are as clear as crystal. There is nothing apparently abnormal in the left eye, external or internal, save the incipient cataract, and nothing apparently abnormal in the right eye save the paralysed pupil. He continues, much evidence at hand goes to support the views of Schoen that all simple and senile cataracts commence as a capsulitis. This produces proliferation of epithelial cells sometimes progressing to hyaline degeneration and perhaps coagulation necrosis. This hypothesis is doubly striking in view of what Forster has shown regarding the effects of slight trituration of the lens capsule with a smooth instrument for the hastening of cataractous development. That such is a fact is easy of demonstration. Weeks moreover has shown that this massage of the lens for hastening opacification, results in adhesion of the capsule and cortex. This indicates capsulitis with proliferating or granulation cells, or perhaps exudative matter. Now the question arises: Does there often exist in the senile eye any condition, pathological or physiological, which could produce even the minute irritation or traumatism of the lens capsule necessary to the production of opacities in the lens proper? The answer is undoubtedly yes, and the answer is fraught with significance. It is an anatomical fact that a portion of the posterior surface of the iris lies and moves in contact with the surface of the anterior capsule of the lens. In youth, and unless certain changes occur which may accompany senility, the iris is a thin and delicate membrane composed of soft, almost impalpable, tissue. The passage of this filmy substance across the surface of the capsule is not harmful. But let the character of its tissues become altered, let them stiffen or roughen, and we have an immediate plausible causation for capsulitis and opacification. For the iris never rests, save while the individual sleeps, and perhaps not even then. There is a constant movement, action and reaction—a continual oscillation, known physiologically as "hippus." This constant grating of a rough or stiffened iris upon the capsule is, without doubt, one of the great causes of cataract. That it has been overlooked is strange, that it should be recognised at once is certain. He thinks it is not a coincidence that in the majority of cataract cases one does not get a full dilatation of the pupil under the influence of mydriatics, but rather that it is accounted for by the thickening or stiffening of the fibres of the iris and not as so often suggested by atrophic conditions. As to the causes of these iritic changes he enumerates arteriosclerosis, gout, rheumatism, Bright's disease, diabetes, and the hyperplasia, the œdema, the rough and sticky exudation, or the inflammatory stiffening that would accompany acute or chronic inflammations of the intraocular structures. Armed with these weapons he represents the relentless iris making a savage attack on the unoffending lens capsule, which eventually succumbs, becomes inflamed, with the unfortunate result of a cataractous condition being set up. Now how is one to prevent all this trouble? Simply by controlling the movements of the iris. This is easy to do with atropin, but atropin, after long continuance, becomes itself a source of irritation to the eye. To sever the fibres of the motor oculi which pass into the lenticular ganglion, and from thence innervate the fibres of the sphincter pupillæ, is too difficult and dangerous an operation to be thought of. Iridectomy is the only remedy left. It is not necessary to do a complete iridectomy; a small piece cut out of the free edge of the iris will take the sphincter, so Jervey prefers to call the operation sphincterectomy. Naturally, the operation must be performed with great skill, so that the capsule of the lens may not be interfered with in its performance, as if this were done—if the lens capsule be pressed on even with the iris as an intervening medium—the very thing we seek to avoid will happen, and the lens become opaque. Still, if the lens does become opaque, an extraction operation can be done later with hopes of a good result.

**Eye Strain and Headache.**—Lucien Howe,[2] at the ninety-seventh annual meeting of the Medical Society of the State of New York, submitted an explanation of the manner in which eye strain caused headache. By the term eye strain he means the pain caused by doing near work in certain people. The pain is referred to the eye itself, the forehead, or other part of the head and even to the shoulders. He wished to prove that the pain was caused by a muscular contraction. An act of accommodation always had associated with it a certain amount of convergence which implied tension on the internal recti and to a certain extent of the superior and inferior recti. The accessory muscles of the forehead and head were called into action when any special effort was required to maintain accommodation, and it was the tension of these muscles which gave rise to the headache. The occipito-frontalis was an important muscle in this respect, and both the anterior and posterior portions were subjected to strain in connection with a special effort to maintain accommodation. This explained frontal and occipital headache.

[1] Med. Rec., Feb. 28, 1903. [2] Lancet, March 7, 1903.

*(To be continued.)*

